# Supplementary material for: Multi-kingdom gut microbiota analyses define COVID-19 severity and post-acute COVID-19 syndrome
Source: Nat Commun. 2022 Nov 10;13:6806. doi: 10.1038/s41467-022-34535-8 (PMC9648868; doi:10.1038/s41467-022-34535-8)
Supplement: Supplementary file 1 — Supplementary information [file 41467_2022_34535_MOESM1_ESM.pdf]

Supplementary Information

Multi-kingdom gut microbiota analyses define COVID-19 severity and post-acute COVID-19 syndrome

Qin Liu, Qi Su, Fen Zhang, Hein M Tun, Joyce Wing Yan Mak, Grace Chung-Yan Lui, Susanna So Shan Ng, Jessica YL Ching, Amy Li, Chenyu Liu, Wenqi Lu, Chun Pan Cheung, David SC Hui, Paul KS Chan, Francis Ka Leung Chan, Siew C Ng*

*Correspondence:
Siew Chien Ng, Department of Medicine and Therapeutics, Faculty of Medicine, The Chinese University of Hong Kong. E-mail: siewchienng@cuhk.edu.hk Tel: (852)3505-1506
Fax: (852) 2647-3852

Supplementary Figures

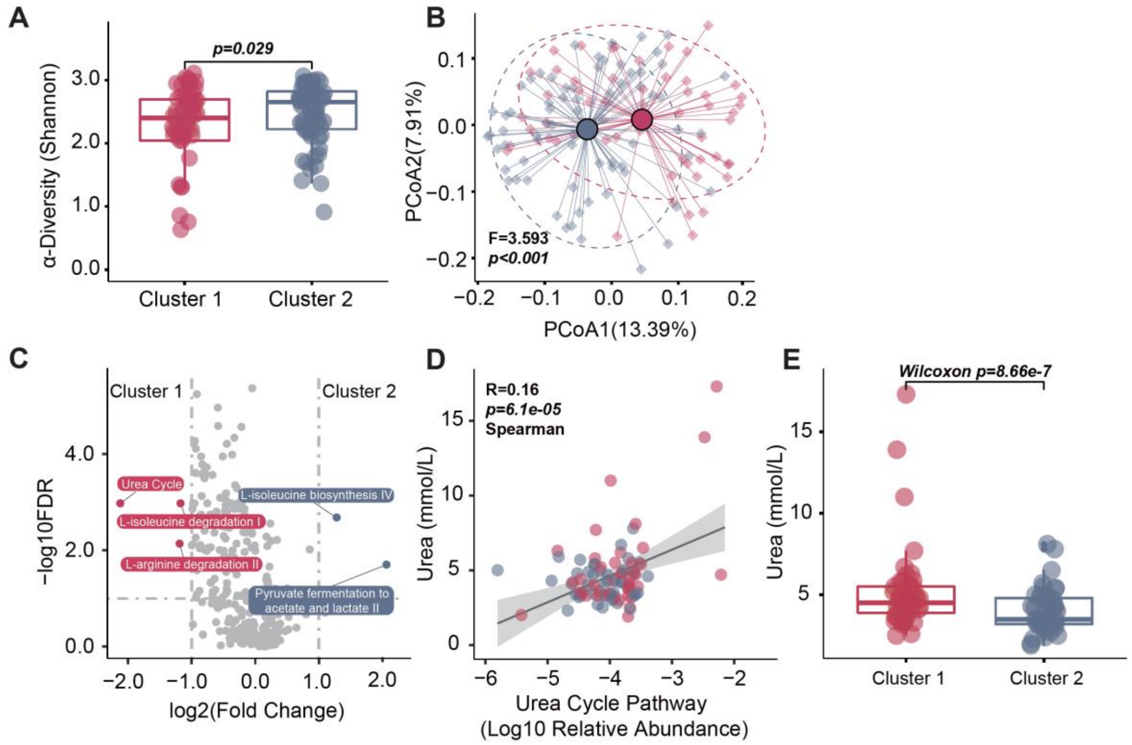

Supplementary Fig. 1

Comparison of gut microbiome composition and microbiome function in different clusters.

Comparison of (A) diversity (Shannon index) (Two-sided Wilcoxon rank-sum test) and (B) PCoA plot illustrating multi-biome diversity in the two clusters (Cluster 1, n=63; Cluster 2, n=70) (PERMANOVA, Adonis test, $p < 0.001$). The circles indicate the cluster of centroids. (C) Microbiome function profiling of patients with COVID-19. Volcano plot showing the effect size log2 fold change (x-axis) and significance (y-axis) of the levels of function between the two clusters. (D) The urea cycle pathway (Log 10 relative abundance) was positively correlated with detected blood urea levels. Error bands reflect the 95%CI. R and P values were calculated by two-sided Spearman Correlation. (E) The blood urea level was significantly higher in Cluster 1. Differences between groups were evaluated by the two-sided Wilcoxon rank-sum test. In A, E, the horizontal line in the boxplot indicates the median value. Box plot lower and upper hinges correspond to the first and third quartiles, upper and lower whiskers represent the highest and lowest values within 1.5 times the interquartile range.

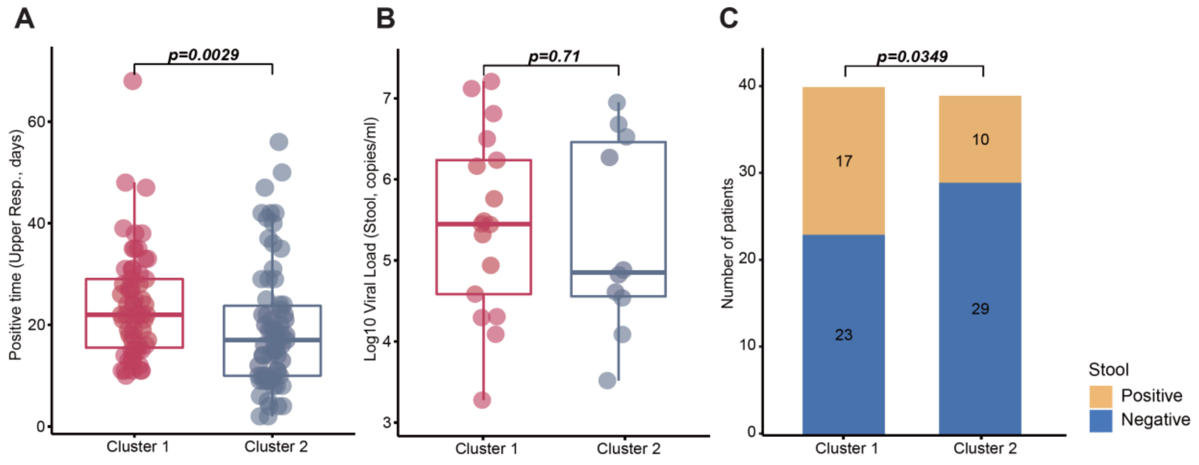

Supplementary Fig. 2

Comparison of positive duration and viral load in respiratory and stool samples of COVID-19 patients

(A) Vial shedding duration in respiratory samples and (B) viral load in stool samples of patients in Cluster 1 was significantly higher than in Cluster 2 (Cluster 1, n=63; Cluster 2, n=70). In A-B, the line in the boxplot indicates the median value. Box plots lower and upper hinges correspond to the first and third quartiles, upper and lower whiskers represent the highest and lowest values within 1.5 times the interquartile range. (C) Positive and negative numbers of SARS-CoV-2 in baseline stool samples detected by RT-qPCR from COVID-19 Patients (n = 79). Differences between groups in A-C were evaluated by the two-sided Wilcoxon rank-sum test

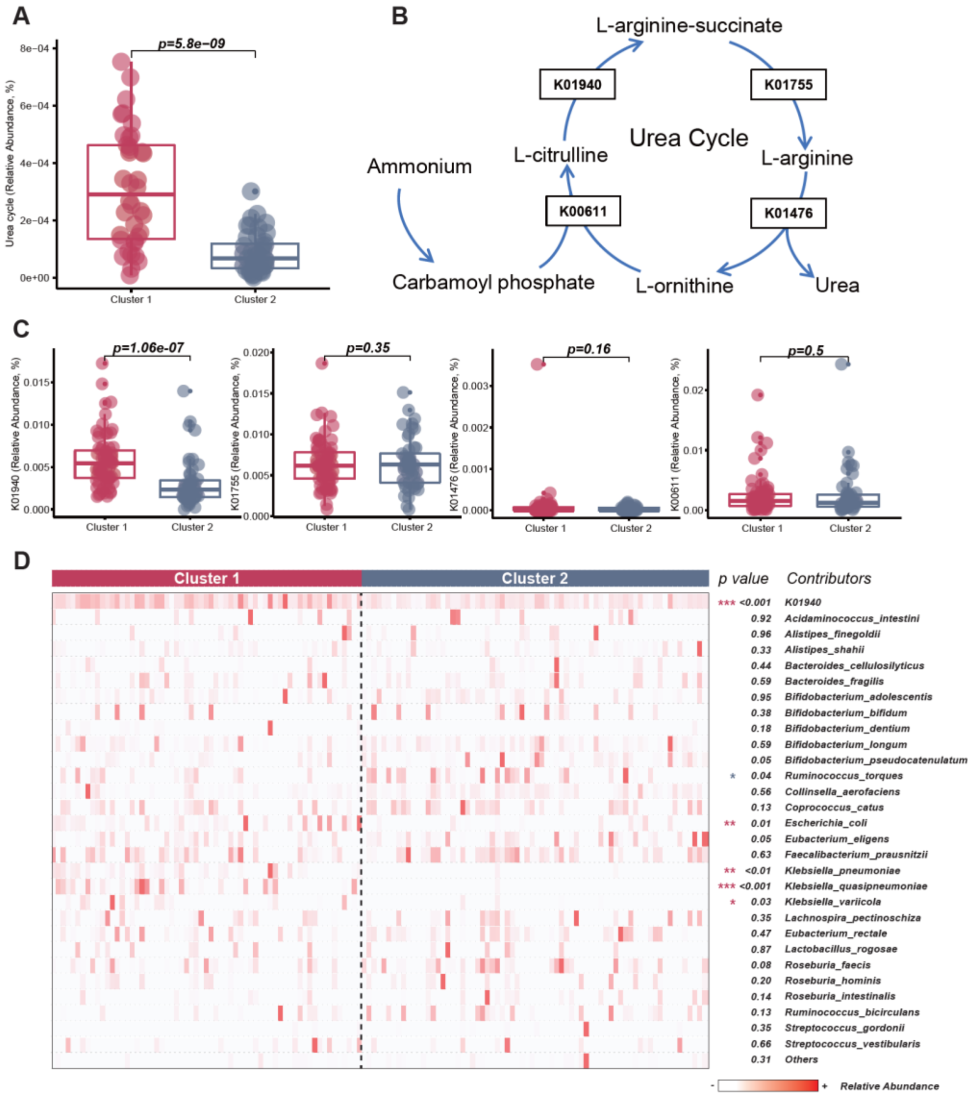

Supplementary Fig. 3 Urea cycle in different clusters. (A) The relative abundance of urea cycle in Cluster 1 and Cluster 2 (Cluster 1, n=63; Cluster 2, n=70). Differences between groups were evaluated by the two-sided Wilcoxon rank-sum test. (B) Schematic diagram of urea cycle showing the enzymes involved and intermediate metabolites. (C) Relative abundance of involved enzymes in Cluster 1 and Cluster 2 (Cluster 1, n=63; Cluster 2, n=70). Differences between groups were evaluated by the two-sided Wilcoxon rank-sum test. (D) Contribution of microbes to the relative abundance of K01940 (argininosuccinate synthase). Differences between groups were evaluated by the two-sided Wilcoxon rank-sum test. In A,C, the horizontal line in the boxplot indicates the median value. Box plots lower and upper hinges correspond to the first and third quartiles, upper and lower whiskers represent the highest and lowest values within 1.5 times the interquartile range.

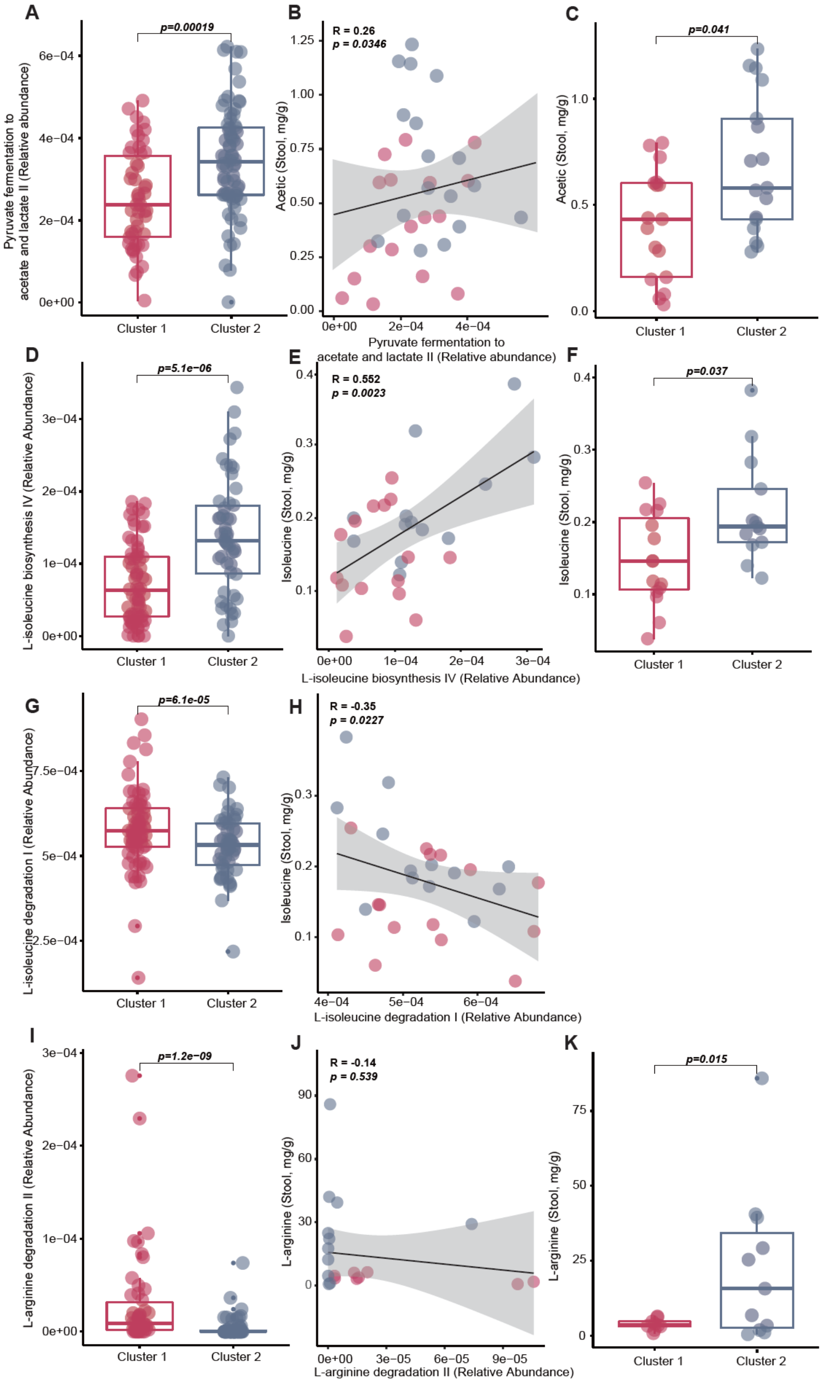

Supplementary Fig. 4 Comparison of metabolite pathway and detected levels in two clusters (Cluster 1, n=63; Cluster 2, n=70), and correlations between pathways and detected metabolites of (A-C) acetic acid. (D-F) L-isoleucine biosynthesis, (G-H) L-isoleucine degradation, and (I-K) L-arginine. In A,C,D,G,I K, differences between groups were evaluated by the two-sided Wilcoxon rank-sum test. The horizontal line in the boxplot indicates the median value. Box plots lower and upper hinges correspond to the first and third quartiles, upper and lower whiskers represent the highest and lowest values within 1.5 times the interquartile range. In B,E,H,J, error bands reflect the 95%CI. R and P values were calculated by two-sided Spearman Correlation.

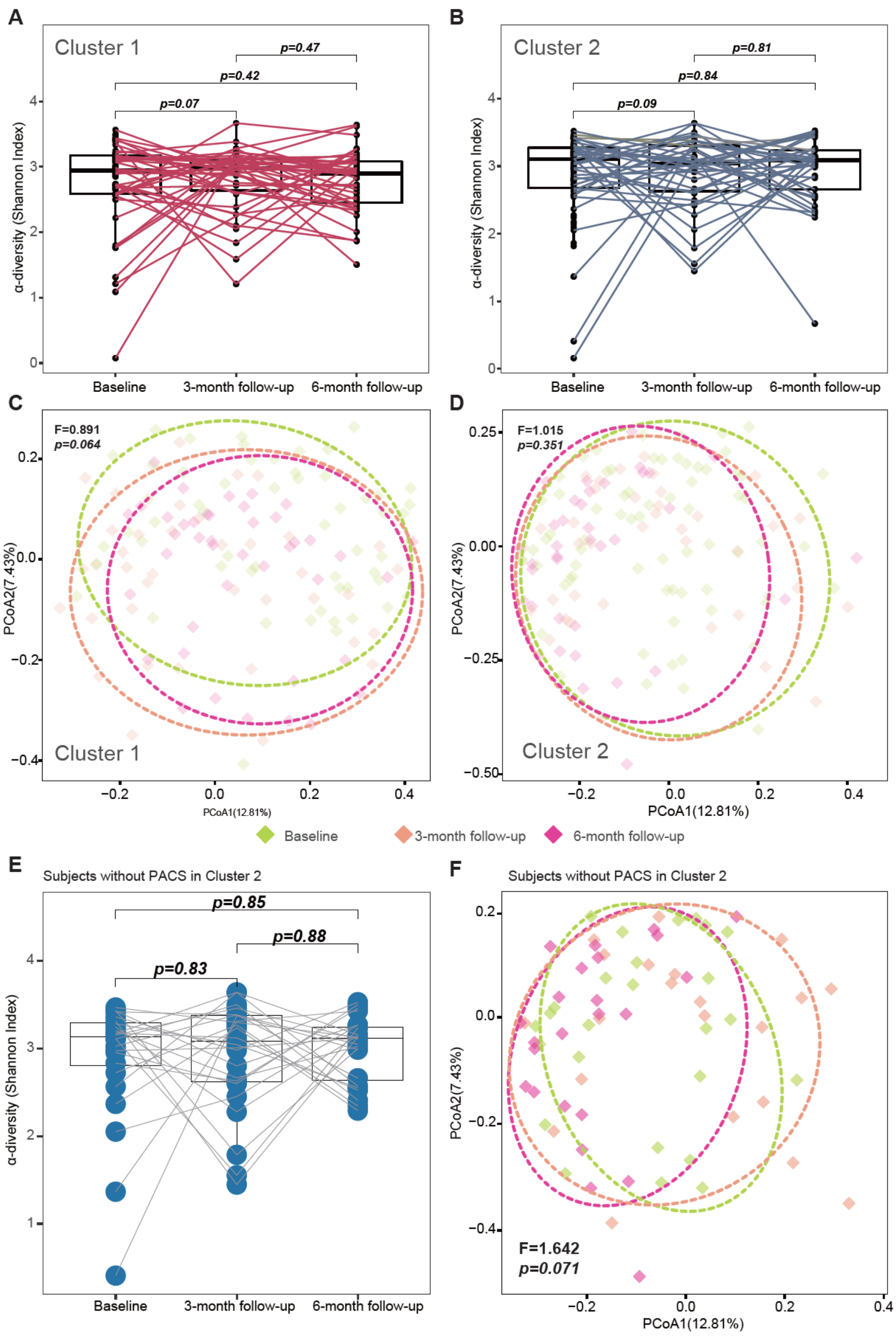

Supplementary Fig. 5 Longitudinal analysis of gut microbiota diversity in patients with COVID-19 at baseline, 3-month follow-up and 6-month follow-up in subjects of Cluster 1 (A) (Cluster 1, n=56 for baseline, n=38 for 3-month, n=42 for 6-month) and Cluster 2 (B) (Cluster 2, n=70 for baseline, n=37 for 3-month, n=37 for 6-month). In A-B, the horizontal line in the boxplot indicates the median value. Box plots lower and upper hinges correspond to the first and third quartiles, upper and lower whiskers represent the highest and lowest values within 1.5 times the interquartile range. Differences between groups in A-B were evaluated by the one-sided Wilcoxon rank-sum test. Principal Coordinate Analysis (PCoA) (PERMANOVA, Adonis test) of multi-biome of patients at baseline, 3-month follow-up, and 6-month follow-up in subjects of Cluster 1 (C) and Cluster 2 (D). Shannon diversity (E) (One-sided Wilcoxon rank-sum test) and PcoA (F) (PERMANOVA, Adonis test) of gut microbiome composition of subjects without PACS in Cluster 2 at baseline, 3-month follow-up, and 6-month follow-up (n=24 for baseline, n=24 for 3-month, n=24 for 6-month).

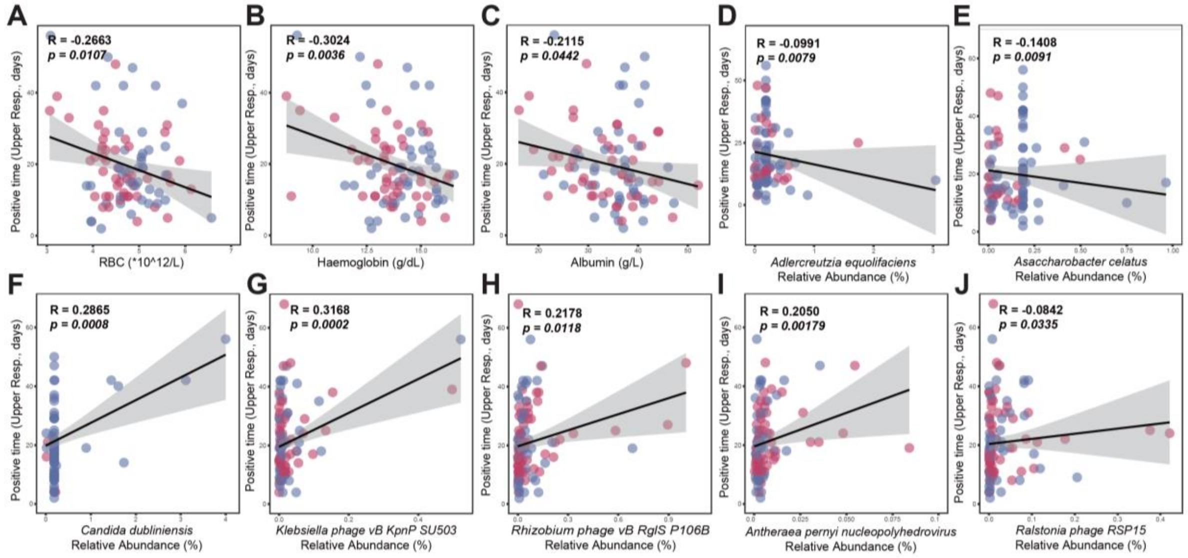

Supplementary Fig. 6

Correlations between viral shedding duration and top 10 contributors (A-J) for the prediction model. Error bands reflect the 95%CI. R and P values were calculated by two-sided Spearman Correlation.

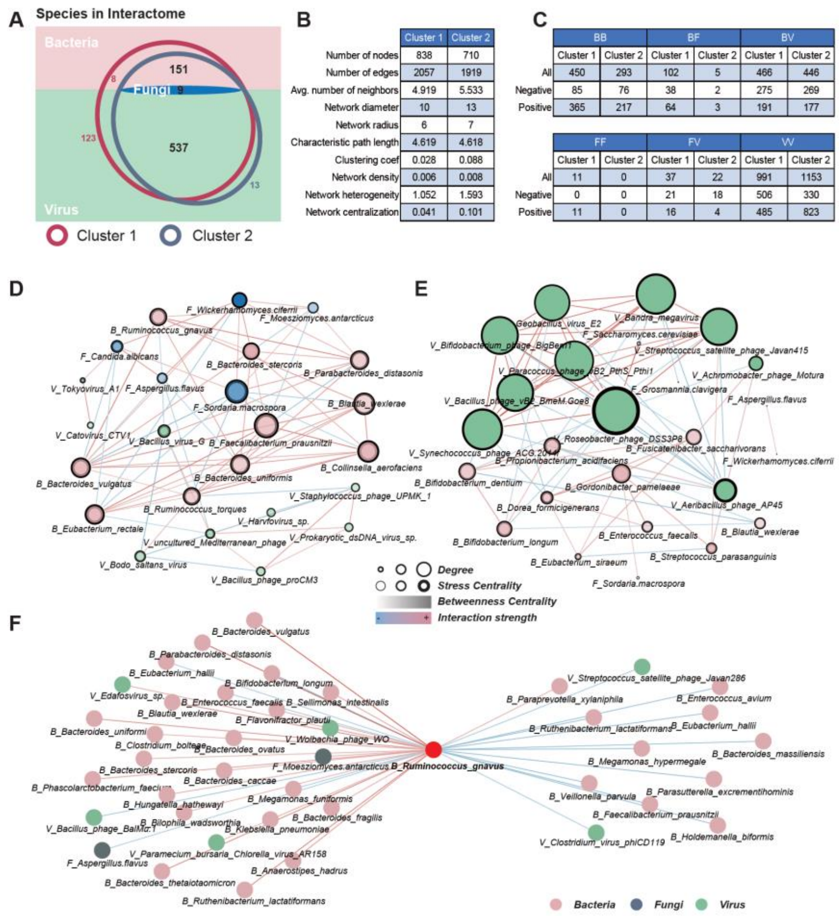

	Cluster 1	Cluster 2
Number of nodes	838	710
Number of edges	2057	1919
Avg. number of neighbors	4.919	5.533
Network diameter	10	13
Network radius	6	7
Characteristic path length	4.619	4.618
Clustering coef	0.028	0.088
Network density	0.006	0.008
Network heterogeneity	1.052	1.593
Network centralization	0.041	0.101

	BB		BF		BV	
	Cluster 1	Cluster 2	Cluster 1	Cluster 2	Cluster 1	Cluster 2
All	450	293	102	5	466	446
Negative	85	76	38	2	275	269
Positive	365	217	64	3	191	177

	FF		FV		VV	
	Cluster 1	Cluster 2	Cluster 1	Cluster 2	Cluster 1	Cluster 2
All	11	0	37	22	991	1153
Negative	0	0	21	18	506	330
Positive	11	0	16	4	485	823

Supplementary Fig. 7 Network analysis of the interactome of COVID-19 patients (A) Venn diagram summarizing the observed interactions of the multi-biome. (B) Summary table listing network characteristics of Cluster 1 and Cluster 2, highlighting the total number of detectable microbes in each network and the total number of interactions. Total and overlapping number of interactions in two clusters. (C) Summary of the positive or negative association numbers between bacteria, fungi and viruses in two clusters. Visualization of interactome interactions between the taxa of bacteria, fungi and viruses in Cluster 1 (D), and Cluster 2 (E). Colored circles represent microbes and lines represent their associated interactions. Circle size (degree) reflects the number of direct interactions for a given microbe. Circle border thickness represents calculated stress centrality for each microbe, while color depth reflects the betweenness centrality (the 'influence') of the microbe in the network. (F) Microbial network graphs in clusters centering on *the Ruminococcus gnavus* node. Microbes directly interacting with *Ruminococcus gnavus* are colored to reflect positive (red) or negative (blue) interactions.
