# Supplementary material for: Multi-kingdom gut microbiota analyses define COVID-19 severity and post-acute COVID-19 syndrome
Source: Nat Commun. 2022 Nov 10;13:6806. doi: 10.1038/s41467-022-34535-8 (PMC9648868; doi:10.1038/s41467-022-34535-8)
Supplement: Supplementary file 3 — Description of Additional Supplementary Files [file 41467_2022_34535_MOESM3_ESM.docx]

**Description of Additional Supplementary Files**

**Supplementary Data 1** Associations between clusters and multi-microbiome by MaAsLin2 (p < 0.05). All p values were adjusted by MaAsLin2 for multiple comparisons using FDR adjustment (Benjamini–Hochberg procedure) and the q-value cut-off of 0.1 was used to identify significant results.

**Supplementary Data 2** PERMANOVA (Adonis test) of the total microbiome community.

**Supplementary Data 3** Microbiome function pathway profile in two clusters. All p values were adjusted by MaAsLin2 for multiple comparisons using FDR adjustment (Benjamini–Hochberg procedure) and the q-value cut-off of 0.1 was used to identify significant results.

**Supplementary Data 4** Comparison of clinical parameters in two clusters. The two-sided Wilcoxon test was used to check the differences between the two clusters.

**Supplementary Data 5** Comparison of cytokines in two clusters. The two-sided Wilcoxon test was used to check the differences between the two clusters.

**Supplementary Data 6** AUC value of different datasets. Differences between groups were evaluated by the two-sided Wilcoxon rank-sum test.

**Supplementary Data 7** Top eleven contributors to the random forest classification model. Differences between groups were evaluated by the two-sided Wilcoxon rank-sum test.

**Supplementary Data 8** Questionnaire used for post-acute COVID-19 symptom assessment.

**Supplementary Data 9** Full list of stool, blood, cytokine, and medical records from patients.
