# Supplementary material for: Multi-kingdom gut microbiota analyses define COVID-19 severity and post-acute COVID-19 syndrome
Source: Nat Commun. 2022 Nov 10;13:6806. doi: 10.1038/s41467-022-34535-8 (PMC9648868; doi:10.1038/s41467-022-34535-8)
Supplement: Supplementary file 11 — Supplementary Data 8 [file 41467_2022_34535_MOESM11_ESM.docx]

**Supplementary Table 9 Questionnaire used for post acute COVID-19 symptom assessment**

| **Symptoms** | **Month 3** | **Month 6** |
| --- | --- | --- |
| Fever |  |  |
| Chills |  |  |
| Cough |  |  |
| Sputum Production |  |  |
| Sore throat |  |  |
| Congested or runny nose |  |  |
| Fatigue |  |  |
| Joint pain |  |  |
| Muscle pain |  |  |
| Shortness of breath |  |  |
| Headache |  |  |
| Dizziness |  |  |
| Nausea |  |  |
| Vomiting |  |  |
| Diarrhoea |  |  |
| Loss of taste |  |  |
| Loss of smell |  |  |
| Abdominal pain |  |  |
| Epigastric pain |  |  |
| Difficulty in concentration |  |  |
| Inability to exercise |  |  |
| Difficulty in sleeping |  |  |
| Anxiety |  |  |
| Sadness |  |  |
| Memory problem |  |  |
| Chest pain |  |  |
| Palpitations |  |  |
| Night sweats |  |  |
| Hair loss |  |  |
| Blurred vision |  |  |
| Any other symptoms |  |  |
