# Supplementary material for: Multi-kingdom gut microbiota analyses define COVID-19 severity and post-acute COVID-19 syndrome
Source: Nat Commun. 2022 Nov 10;13:6806. doi: 10.1038/s41467-022-34535-8 (PMC9648868; doi:10.1038/s41467-022-34535-8)
Supplement: Supplementary file 13 — Reporting Summary [file 41467_2022_34535_MOESM13_ESM.pdf]

nature portfolio

Corresponding author(s): Siew C Ng

Last updated by author(s): 25/10/22

Reporting Summary

Nature Portfolio wishes to improve the reproducibility of the work that we publish. This form provides structure for consistency and transparency in reporting. For further information on Nature Portfolio policies, see our Editorial Policies and the Editorial Policy Checklist.

Statistics

For all statistical analyses, confirm that the following items are present in the figure legend, table legend, main text, or Methods section.

n/a	Confirmed	
☐	☒	The exact sample size (*n*) for each experimental group/condition, given as a discrete number and unit of measurement
☐	☒	A statement on whether measurements were taken from distinct samples or whether the same sample was measured repeatedly
☐	☒	The statistical test(s) used AND whether they are one- or two-sided<br>*Only common tests should be described solely by name; describe more complex techniques in the Methods section.*
☐	☒	A description of all covariates tested
☐	☒	A description of any assumptions or corrections, such as tests of normality and adjustment for multiple comparisons
☐	☒	A full description of the statistical parameters including central tendency (e.g. means) or other basic estimates (e.g. regression coefficient) AND variation (e.g. standard deviation) or associated estimates of uncertainty (e.g. confidence intervals)
☐	☒	For null hypothesis testing, the test statistic (e.g. *F*, *t*, *r*) with confidence intervals, effect sizes, degrees of freedom and *P* value noted<br>*Give P values as exact values whenever suitable.*
☒	☐	For Bayesian analysis, information on the choice of priors and Markov chain Monte Carlo settings
☐	☒	For hierarchical and complex designs, identification of the appropriate level for tests and full reporting of outcomes
☐	☒	Estimates of effect sizes (e.g. Cohen's *d*, Pearson's *r*), indicating how they were calculated

Our web collection on statistics for biologists contains articles on many of the points above.

Software and code

Policy information about availability of computer code

Data collection	No software was used for sample collection
Data analysis	Continuous variables were expressed in median (interquartile range) whereas categorical variables were presented as numbers (percentage). Qualitative and quantitative differences between subgroups were analysed using chi-squared or Fisher's exact tests for categorical parameters and Wilcoxon rank-sum test for continuous parameters, as appropriate. Odds ratio and adjusted odds ratio (aOR) with 95% confidence interval (CI) were estimated using logistic regression to examine clinical parameters associated with the development of PACS. The site by species counts and relative abundance tables were input into R V.3.5.1 for statistical analysis. Principal Coordinates Analysis (PCoA) was used to visualise the clustering of samples based on their species-level compositional profiles. Associations between gut community composition and patients' parameters were assessed using permutational multivariate analysis of variance (PERMANOVA). Associations of specific microbial species with patient parameters were identified using the linear discriminant analysis effect size (LEfSe) and the multivariate analysis by linear models (MaAsLin2) statistical frameworks implemented in the Huttenhower Lab Galaxy instance (http:// huttenhower.sph.harvard.edu/galaxy/). PCoA, PERMANOVA and Procrustes analysis are implemented in the vegan R package V.2.5–7.

For manuscripts utilizing custom algorithms or software that are central to the research but not yet described in published literature, software must be made available to editors and reviewers. We strongly encourage code deposition in a community repository (e.g. GitHub). See the Nature Portfolio guidelines for submitting code & software for further information.

Data

Policy information about availability of data

All manuscripts must include a data availability statement. This statement should provide the following information, where applicable:

- Accession codes, unique identifiers, or web links for publicly available datasets
- A description of any restrictions on data availability
- For clinical datasets or third party data, please ensure that the statement adheres to our policy

Raw sequence data are available in the Sequence Read Archive (SRA) under BioProject accession PRJNA876804

Field-specific reporting

Please select the one below that is the best fit for your research. If you are not sure, read the appropriate sections before making your selection.

☐ Life sciences ☐ Behavioural & social sciences ☐ Ecological, evolutionary & environmental sciences

For a reference copy of the document with all sections, see nature.com/documents/nr-reporting-summary-flat.pdf

Life sciences study design

All studies must disclose on these points even when the disclosure is negative.

Sample size	Given the exploratory nature of the study, no formal power calculation was performed.
Data exclusions	Patients who fulfilled the following criteria were eligible for analyses: (i) 18 -70 years of age, (ii) no antibiotic therapy before at least 6 months, during and 6 months after acute infection of SARS-CoV-2 (iii) no gastrointestinal symptoms during acute infection. Written informed consent was obtained from all patients. Dietary data were documented for all COVID-19 patients during the time of hospitalisation (whereby standardised meals were provided by the hospital catering service of each hospital) and individuals with special eating habits such as vegetarians were excluded.
Replication	The study was not formally replicated.
Randomization	This is an observation study without treatment groups.
Blinding	This is an observation study with no blinding as there was no treatment groups.

Behavioural & social sciences study design

All studies must disclose on these points even when the disclosure is negative.

Study description	*Briefly describe the study type including whether data are quantitative, qualitative, or mixed-methods (e.g. qualitative cross-sectional, quantitative experimental, mixed-methods case study).*
Research sample	*State the research sample (e.g. Harvard university undergraduates, villagers in rural India) and provide relevant demographic information (e.g. age, sex) and indicate whether the sample is representative. Provide a rationale for the study sample chosen. For studies involving existing datasets, please describe the dataset and source.*
Sampling strategy	*Describe the sampling procedure (e.g. random, snowball, stratified, convenience). Describe the statistical methods that were used to predetermine sample size OR if no sample-size calculation was performed, describe how sample sizes were chosen and provide a rationale for why these sample sizes are sufficient. For qualitative data, please indicate whether data saturation was considered, and what criteria were used to decide that no further sampling was needed.*
Data collection	*Provide details about the data collection procedure, including the instruments or devices used to record the data (e.g. pen and paper, computer, eye tracker, video or audio equipment) whether anyone was present besides the participant(s) and the researcher, and whether the researcher was blind to experimental condition and/or the study hypothesis during data collection.*
Timing	*Indicate the start and stop dates of data collection. If there is a gap between collection periods, state the dates for each sample cohort.*
Data exclusions	*If no data were excluded from the analyses, state so OR if data were excluded, provide the exact number of exclusions and the rationale behind them, indicating whether exclusion criteria were pre-established.*
Non-participation	*State how many participants dropped out/declined participation and the reason(s) given OR provide response rate OR state that no participants dropped out/declined participation.*

Randomization	*If participants were not allocated into experimental groups, state so OR describe how participants were allocated to groups, and if allocation was not random, describe how covariates were controlled.*

Ecological, evolutionary & environmental sciences study design

All studies must disclose on these points even when the disclosure is negative.

Study description	*Briefly describe the study. For quantitative data include treatment factors and interactions, design structure (e.g. factorial, nested, hierarchical), nature and number of experimental units and replicates.*
Research sample	*Describe the research sample (e.g. a group of tagged Passer domesticus, all Stenocereus thurberi within Organ Pipe Cactus National Monument), and provide a rationale for the sample choice. When relevant, describe the organism taxa, source, sex, age range and any manipulations. State what population the sample is meant to represent when applicable. For studies involving existing datasets, describe the data and its source.*
Sampling strategy	*Note the sampling procedure. Describe the statistical methods that were used to predetermine sample size OR if no sample-size calculation was performed, describe how sample sizes were chosen and provide a rationale for why these sample sizes are sufficient.*
Data collection	*Describe the data collection procedure, including who recorded the data and how.*
Timing and spatial scale	*Indicate the start and stop dates of data collection, noting the frequency and periodicity of sampling and providing a rationale for these choices. If there is a gap between collection periods, state the dates for each sample cohort. Specify the spatial scale from which the data are taken*
Data exclusions	*If no data were excluded from the analyses, state so OR if data were excluded, describe the exclusions and the rationale behind them, indicating whether exclusion criteria were pre-established.*
Reproducibility	*Describe the measures taken to verify the reproducibility of experimental findings. For each experiment, note whether any attempts to repeat the experiment failed OR state that all attempts to repeat the experiment were successful.*
Randomization	*Describe how samples/organisms/participants were allocated into groups. If allocation was not random, describe how covariates were controlled. If this is not relevant to your study, explain why.*
Blinding	*Describe the extent of blinding used during data acquisition and analysis. If blinding was not possible, describe why OR explain why blinding was not relevant to your study.*

Did the study involve field work? ☐ Yes ☐ No

Field work, collection and transport

Field conditions	*Describe the study conditions for field work, providing relevant parameters (e.g. temperature, rainfall).*
Location	*State the location of the sampling or experiment, providing relevant parameters (e.g. latitude and longitude, elevation, water depth).*
Access & import/export	*Describe the efforts you have made to access habitats and to collect and import/export your samples in a responsible manner and in compliance with local, national and international laws, noting any permits that were obtained (give the name of the issuing authority, the date of issue, and any identifying information).*
Disturbance	*Describe any disturbance caused by the study and how it was minimized.*

Reporting for specific materials, systems and methods

We require information from authors about some types of materials, experimental systems and methods used in many studies. Here, indicate whether each material, system or method listed is relevant to your study. If you are not sure if a list item applies to your research, read the appropriate section before selecting a response.

Materials & experimental systems

n/a	Involved in the study	
☒	☐	Antibodies
☒	☐	Eukaryotic cell lines
☒	☐	Palaeontology and archaeology
☒	☐	Animals and other organisms
☐	☒	Human research participants
☐	☒	Clinical data
☒	☐	Dual use research of concern

Methods

n/a	Involved in the study	
☒	☐	ChIP-seq
☒	☐	Flow cytometry
☒	☐	MRI-based neuroimaging

Human research participants

Policy information about studies involving human research participants

Population characteristics	Participants were recruited and consented under Research Ethics Committee (REC) no. 2020.076 and all subjects provided informed consent. This is a cross-sectional and prospective cohort study involving 133 patients with a confirmed diagnosis of COVID-19 (defined as positive RT-PCR test for SARS-CoV-2 in nasopharyngeal swab, deep throat saliva, sputum or tracheal aspirate) hospitalised at three regional hospitals (Prince of Wales Hospital, United Christian Hospital and Yan Chai Hospital) in Hong Kong, China between 13 March 2020 and 27 Jan 2021, followed-up to six months.
Recruitment	Recruitment took place between March 2020 and Aug 2021.
Ethics oversight	Participants were recruited and consented under Research Ethics Committee (REC) no. 2020.076 and all subjects provided informed consent.

Note that full information on the approval of the study protocol must also be provided in the manuscript.

Clinical data

Policy information about clinical studies

All manuscripts should comply with the ICMJEguidelines for publication of clinical research and a completedCONSORT checklist must be included with all submissions.

Clinical trial registration	NA
Study protocol	The study protocol was approved by Research Ethics Committee (REC) no. 2020.076 .
Data collection	Recruitment included three regional hospitals (Prince of Wales Hospital, United Christian Hospital and Yan Chai Hospital) in Hong Kong, China
Outcomes	Our analysis was hypothesis generating.We performed an integrated analysis using 296 fecal metagenomes, 1,378 severe acute respiratory syndrome coronavirus 2 (SARS-CoV-2) viral load of respiratory samples and clinical features of 133 patients with COVID-19 prospectively followed-up to 6 months.Our cross-sectional and prospective multi-omics analysis revealed several new insights of the role of host and microbial factors in COVID-19 severity and its long-term complication
